# Scaling HIV self‐testing to improve health equity among cisgender gay, bisexual and other men who have sex with men, trans and gender‐diverse people in the United Kingdom: An HIV self‐testing implementation action framework

**DOI:** 10.1111/hiv.70229

**Published:** 2026-04-22

**Authors:** T. Charles Witzel, Peter Weatherburn, Fiona M. Burns, Isaac Y.‐H. Chu, Phil Samba, Alison J. Rodger

**Affiliations:** ^1^ Institute for Global Health, University College London London UK; ^2^ Faculty of Public Health and Policy London School of Hygiene and Tropical Medicine London UK; ^3^ Royal Free London NHS Foundation Trust London UK; ^4^ The Love Tank CIC London UK

**Keywords:** differentiated service delivery, Europe, gay, bisexual and other men who have sex with men, HIV self‐testing, implementation science, person‐centred care, trans and gender‐diverse people, UK

## Abstract

**Background:**

HIV self‐testing (HIVST) is feasible, highly acceptable and can increase HIV testing uptake/frequency without harm while also reducing costs. Despite HIVST's benefits, UK service provision has been sporadic partially due to policymakers and commissioners' reluctance over concerns about linkage to care/surveillance, which ultimately reduces user choice.

**Methods:**

We synthesized current evidence and in a multidisciplinary collaboration developed an Implementation Action Framework to facilitate and promote person‐centred HIVST provision, focusing on reducing health inequalities among gay, bisexual and other men who have sex with men (GBMSM), trans and gender‐diverse people. We do this by exploring implementation context, establishing a standard level of support for HIVST interventions and outlining intervention adaptations to support priority subgroups in the United Kingdom.

**Results and Discussion:**

A large body of published evidence demonstrates that HIVST is feasible to deliver, highly acceptable, easy to perform and can make a meaningful difference to HIV testing behaviours among GBMSM, trans and gender‐diverse people without leading to substantial harm or decreases in linkage to care. Synthesizing 29 publications from the United Kingdom or other high‐income settings, we demonstrate that the UK implementation context can be harnessed by emphasizing the need to expand testing to meet the 2030 HIV elimination goals. Concerns around linkage to care/surveillance can be addressed by highlighting successful linkage approaches and the importance of respecting patient autonomy. We establish a minimum standard level of support for HIVST interventions, including a results reporting system with a user opt‐out option, clear information on linkage to care, inclusion of a helpline and guidance to link to clinical follow‐up for those who report reactive HIVST results. To address health inequalities, person‐centred intervention adaptations include innovative approaches to HIVST delivery to reach underserved groups, provision of additional tests for other sexually transmitted infections (STIs), targeted demand generation and tailored support for the most marginalized.

**Conclusion:**

Person‐centred HIVST is critical to improving HIV testing uptake by responding to, and ultimately reducing, entrenched health inequalities in the United Kingdom. Our Framework provides a person‐centred roadmap for implementing HIVST across a range of high‐income settings.

## BACKGROUND

The HIV epidemic among gay, bisexual and other men who have sex with men (GBMSM), trans and gender‐diverse people in the United Kingdom remains a significant public health challenge. Although there have been substantial reductions in HIV incidence since 2015, these are not shared equitably [1]. While White GBMSM have experienced falling numbers of HIV diagnoses (517 in 2020 to 446 in 2024), GBMSM from ethnic minority communities remain disproportionately impacted, with diagnoses increasing nearly 50% over the same period (194 to 285) [2]. Further, trans people are twice as likely to be diagnosed with HIV late compared with cisgender people [3]. Without targeted action to expand HIV testing in these key populations, health inequalities are likely to increase further.

Rapid diagnosis of HIV has benefits not just for individual health, but for onward transmission of HIV [4, 5, 6] as successful antiretroviral therapy (ART) means an individual cannot pass on HIV sexually [7, 8, 9]. The ‘Test and Treat’ approach (aligned with the UNAIDS global 95–95–95 targets) provided a critical impetus to rapidly and sustainably expand testing [10], though recent evidence from modelling suggests the likelihood of the United Kingdom achieving their target of less than 50 new diagnoses in GBMSM in England by 2030 on the current trajectory is zero [11].

The use of HIV rapid diagnostic tests (RDTs) in the United Kingdom expanded from the mid‐2000s with use in community‐based testing programmes which extended testing to community settings, bars and nightclubs. This aimed to increase testing access and to reduce barriers relating to limited accessibility of clinics and concerns around stigma and homophobia [12, 13]. HIV self‐sampling (HIVSS) is where an individual collects a blood sample themselves and returns it to a laboratory for processing. Available in the United Kingdom from 2012, this brought HIV testing into the home. Free HIVSS is currently available in most local authorities in England and all areas of Wales. However, HIVSS sample return rates remain between 50% and 60% [14, 15], likely leading to missed opportunities for timely diagnosis.

HIV self‐testing (HIVST) allows an individual to test themselves for HIV using an RDT. The individual takes their own sample (either blood or saliva), processes their test and reads their own result. This approach was first recommended by the World Health Organization (WHO) as an additional testing option in 2016, a recommendation that was strengthened in 2019 [16, 17]. Since 2024, the WHO has recommended HIVST be used to support HIV pre‐exposure prophylaxis (PrEP) service delivery [18], indicating an expanding role for this new technology. HIVST also provides greater privacy, which can be particularly important for some populations.

Another critical benefit of HIVST is the flexibility and adaptability of the intervention. Indeed, self‐testing can be delivered in a huge range of ways with a variety of supportive options and packaged with tests for additional infections [19, 20]. This means that HIVST can be tailored for specific populations through person‐centred approaches, with the key goal of improving health equity. HIVST was legalized in the United Kingdom in 2014 and recommended in UK national guidelines for the first time in 2020 [21], however, as of 2026, progress towards implementing routine access to HIVST in the United Kingdom has been limited.

The aim of developing this HIVST Implementation Action Framework was to facilitate and promote HIVST service delivery in the United Kingdom based upon a clear review of available evidence with the key goal of improving health equity among GBMSM, trans and gender‐diverse people. The Implementation Framework outlined here, and published elsewhere (see Witzel et al. [22]), is intended to be used by those considering or planning to implement HIVST in the United Kingdom (or other high‐income setting) among these populations, including those in commissioning bodies, policymakers and the voluntary and community sector. We envisage widespread HIVST implementation as providing additional choice for those who experience barriers to testing while also supporting multifaceted sexual health service provision, including clinic‐based sexual health services which are vital to meet the needs of many. This Framework focuses only on GBMSM, trans and gender‐diverse populations due to the lack of research on HIVST in other priority populations in the United Kingdom—especially Black African heterosexual communities. It is a critical priority to develop this evidence base as these groups are especially likely to be left behind by HIV testing and prevention innovations.

## METHODS

### Evidence synthesis

To develop this Implementation Action Framework, we synthesized key evidence from a range of sources. This included a scoping review of published literature detailing HIVST implementation and evaluation, systematic reviews, conference abstracts and reports from community and voluntary sector organizations. We did not use predefined inclusion and exclusion criteria. Rather, we extended a framework previously developed (see Witzel [23]). We then actively searched for evidence to fill gaps and consolidate understanding from a broad range of published literature. Where possible, evidence was drawn from the United Kingdom, looking to comparable high‐income settings where gaps remained. This was done by TCW (updated to 2025) and reviewed within the team.

The evidence was synthesized by mapping findings from the literature review onto Consolidated Framework for Implementation Research (CFIR) domains (see below) [24, 25]. Throughout the Framework, we present empirical findings from our review alongside recommendations based on this evidence and our stakeholder engagement, our implementation expertise and clinical decision‐making.

Developing the standard level of support involved reviewing previous implementation initiatives [26, 27, 28, 29, 30, 31] alongside intensive discussions within the immediate team on balancing feasibility of delivery alongside acceptability to beneficiaries, commissioners and policy makers, who may have divergent interests. We sought to balance how best to support potential beneficiaries while addressing provider concerns, especially around linkage to care. We further refined our approach through community and stakeholder engagement, described below.

### Consolidated Framework for Implementation Research

We used CFIR (version 1.0) to structure our Implementation Action Framework. CFIR highlights how successful implementation and translation of interventions works best if attention is paid to five key domains [24, 25, 32, 33]. Firstly, CFIR focuses on the intervention (in our case, HIVST interventions generally), which contains all characteristics of the intervention that is being implemented, acknowledging that many need adaptations to work optimally [24]. This is divided into intervention core (the elements of the intervention unlikely to change) and periphery (all elements which can be adapted). Then, CFIR explores the role of context (inner and outer setting) in shaping intervention delivery, especially the structural, political, economic and social contexts in which an intervention is implemented [24]. For our purposes, the inner setting refers to meso actors—for example, local commissioning bodies, sexual health clinics and voluntary sector organizations—whereas the outer setting includes the health system generally and the policy environment. CFIR also explores how individuals involved shape implementation. CFIR typically looks at both intervention beneficiaries and those delivering the intervention; for this framework, we focus on the latter, acknowledging that acceptability and need for HIVST is high among the intended target population. Finally, CFIR explores the process of implementation itself, including planning, executing and evaluating [24].

### Community and stakeholder engagement

Throughout Framework development, we conducted participant and public involvement (PPI) with members of affected communities (GBMSM and trans people) and key stakeholders in the HIV response.

We held three meetings with members of affected communities recruited through the personal and professional networks of our PPI co‐investigator (author PS). Meetings took place after hours in private rooms in a community organization and a central London University. Participants were provided with refreshments and compensated £40 for their time. Support resources were available if needed.

The first PPI meeting explored the utility of additional analyses planned to support the Implementation Framework. The second reviewed these analyses, and the third focused on refining the Framework. Discussions did not include questions about health behaviours and instead focused on participants' general views.

We also established a Community and Policy Engagement Group made up of stakeholders from clinical, voluntary, policy and commissioning organizations working in HIV testing (including HIVST and HIVSS) in England and Wales. Members contributed by ensuring the evidence review was comprehensive, recommended key studies and helped develop a detailed understanding of the UK HIVST implementation context. An early meeting refined the Framework's scope and analysis while later sessions shaped our recommendations to ensure relevance for the populations and individuals we seek to reach.

Ethical approval was not required for PPI activities or for the Community and Policy Engagement Group as these meetings focused on feedback about our analyses and recommendations. Nevertheless, we established confidentiality ground rules to ensure discussions took place in a safe and open manner.

## RESULTS AND DISCUSSION

### Published literature

The evidence base globally and in England and Wales has evolved rapidly over the last decade. HIVST provides an additional testing choice that reduces barriers to use of conventional services such as stigma and opportunity cost [34, 35]. There is a large body of published evidence demonstrating that HIVST is feasible to deliver, highly acceptable, easy to perform and can make a meaningful difference to HIV testing behaviours among GBMSM, trans and gender‐diverse people without leading to substantial harm or decreases in linkage to care [19, 20, 26, 27, 34, 36, 37, 38, 39, 40, 41]. Table 1 presents 29 included studies and Table 2 summarizes the primary research findings identified, exploring key themes by: implementation context; intervention acceptability particularly in key subgroups considered at increased risk of HIV acquisition; feasibility; values and preferences; and HIVST outcomes.

**TABLE 1:** Overview of included studies.

References	Year	Author	Study type	Data collection	Location	Study population	Aim
[57]	2015	Figueroa et al.	Literature review	Literature review (1995–2014)	Worldwide	Key populations	Focus on the acceptability, values and preferences on HIVST among key populations
[58]	2016	Gibson et al.	Demonstration project	Record analysis	United Kingdom	General population	Determine feasibility and acceptability of HIV home/self‐testing
[37]	2016	Witzel et al.	Qualitative	Focus group discussion	United Kingdom	GBMSM	Explore the acceptability, preferences and concerns about HIVST
[59]	2016	Witzel et al.	Quantitative	Survey	United Kingdom	MSM (both cis and trans)	Identify which groups of MSM are less likely to have tested for HIV and their preferences for future tests
[41]	2017	Flowers et al.	Mixed methods	Survey Focus group discussion	United Kingdom	Cis‐MSM	Explore preparedness for the HIVST among MSM and professionals in HIV care
[31]	2017	Brady et al.	Demonstration project	Record analysis	United Kingdom	HIVST users	Evaluate the feasibility and acceptability of HIV self‐testing in a large‐scale pilot
[56]	2017	Witzel et al.	Qualitative	Personal interview	United Kingdom	Service providers and commissioners	Understand the perspectives of key informants on the implementation of HIVST
[60]	2017	Witzel et al.	Qualitative	Focus group discussion	United Kingdom	MSM (both cis and trans)	Understand how HIVST compliments existing testing strategies considered or adopted by MSM
[38]	2018	Saunders et al.	Demonstration project	Record analysis	United Kingdom	Service users at a sexual health clinic in London	Determine whether self‐testing‐naïve individuals can correctly perform and interpret HIVST
[29]	2019	Baraitser et al.	Quantitative evaluation	Record analysis	United Kingdom	Users of a website offering free HIV tests	Measure the type of test chosen (HIVST or HIV self‐sampling) and reasons for choices
[52]	2019	Vera et al.	Demonstration project	Survey and Record analysis	United Kingdom	Potential users of HIVST	Evaluate the acceptability and feasibility of using vending machine to distribute HIVST in communities
[27]	2019	Witzel et al.	Mixed methods	Survey and personal interviews	United Kingdom	GBMSM and trans women	Assess the feasibility of recruiting SELPHI trial participants and kit receivers' acceptability of HIVST
[34]	2019	Witzel et al.	Qualitative	Personal interview	United Kingdom	GBMSM	Explore how self‐testers experience HIVST and the implications for intervention scale‐up
[61]	2020	Rodger et al.	Trial	Trial surveys	United Kingdom	MSM (both cis and trans)	Report the frequency of previous HIV testing at baseline in MSM enrolling in a randomized controlled trial
[19]	2020	Witzel et al.	Systematic review	Literature review (2006–2019)	Worldwide	Key populations	Understand which service delivery models of HIVST are effective for key populations
[40]	2020	Witzel et al.	Qualitative	Personal interview	United Kingdom	GBMSM	Explore how HIVST interventions were experienced and the mechanisms of action leading to impact
[62]	2021	Middleton et al.	Qualitative	Personal interview and Focus group discussion	United Kingdom	People with mild learning disabilities	Explore barriers and facilitators to correct use of self‐sampling kits for blood‐borne virus and bacterial STI
[36]	2021	Witzel et al.	Mixed methods	Survey and personal interviews	United Kingdom	Trans people	Describe key HIVST outcomes and HIVST acceptability for trans people
[35]	2022	Nicholls et al.	Qualitative	Personal interview	United Kingdom	Asian, Black and Latin American GBMSM	Explore participants' experiences in accessing the gay scene, HIV testing services and preferred adaptation of HIVST
[26]	2022	Rodger et al.	Trial	National surveillance, trial surveys	United Kingdom	MSM (both cis and trans)	Assess whether the offer of a single, free HIVST kit led to increased HIV diagnoses with linkage to care
[44]	2023	Howells et al.	Demonstration project	Record analysis	United Kingdom	HIVST users	Compare the uptake of HIVST to HIV self‐sampling among users of online‐ordered free tests
[11]	2023	Cambiano et al.	Quantitative	Modelling	United Kingdom	GBMSM	Understand the contribution of different interventions in reducing HIV incidence
[63]	2023	Nadarzynski et al.	Quantitative	Survey	United Kingdom	Men and gender‐diverse people who have sex with men	Identify the characteristics of respondents requiring sexual health promotion and clinical support
[39]	2023	Witzel et al.	Mixed methods	Survey and personal interviews	United Kingdom	MSM (both cis and trans)	Understand the relationship between HIVST and harm in SELPHI, the largest randomized trial of HIVST in the United Kingdom
[28]	2024	Dunn et al.	Trial	National Surveillance, Trial surveys		GBMSM and trans people	Describe whether providing regular self‐testing kits to HIV‐seronegative MSM, deemed to be at higher risk of acquiring HIV, reduced the time to the clinically confirmed diagnosis of the infection.
[49]	2024	Chu et al.	Qualitative	Personal interview	United Kingdom	GBMSM and trans women	Develop a contextual understanding of individuals' social networks and HIV testing needs
[53]	2024	Chu et al.	Qualitative	Personal interview	United Kingdom	GBMSM and trans women	Explore the perspectives of multiply marginalized participants on whether HIVST might increase their uptake of HIV testing
[43]	2024	Gobin et al.	Demonstration project	Observations and survey	United Kingdom	HIVST users	Investigate the acceptability and uptake of publicly available machines dispensing STI and HIV testing kits.
[64]	2024	Palich et al.	Qualitative	Personal interview	United Kingdom	GBMSM and trans people	Explore how people reporting non‐consensual sex accessed health care services and how this experience influenced subsequent HIV testing

Abbreviations: GBMSM, gay, bisexual and other men who have sex with men; HIVST, HIV self‐testing; MSM, men who have sex with men; SELPHI, HIV self‐testing public health intervention; STI, sexually transmitted infection.

**TABLE 2:** Key evidence supporting HIV self‐testing in England and Wales.

Area	Finding	Sources
HIVST implementation context		
Public health environment	‘Zero’ chance of reaching the 2030 HIV transmission target of fewer than 50 infections among GBMSM in England. A combined substantial increase in HIV testing (by 30%) and an increase in PrEP provision could avert 34% of new HIV infections. This would require a 16% reduction in the cost of delivery of testing to remain cost‐effective. Self‐testing has a key role in doing this while keeping costs low.	[11]
Organizational contexts	HIVST broadly acceptable to stakeholders, including clinical and community‐based organization staff. However, some have concerns about support for individuals self‐testing.	[56]
Acceptability, feasibility and values and preferences		
Acceptability	HIVST is highly acceptable to a diverse range of GBMSM, trans and gender‐diverse people.	[27, 35, 36, 37, 59, 60]
	HIVST may be a supplementary testing option for most GBMSM, unless they have psychosocial or geographic barriers to existing services.	[37, 60]
	For GBMSM from minority ethnic communities, HIVST provides opportunity to avoid clinic waiting rooms and staff perceived to be potential sources of confidentiality breaches.	[35]
	HIVST enables trans people to avoid testing in sexual health clinics which can be sites of discrimination and can increase dysphoria.	[36]
Feasibility	HIVST is feasible to implement for GBMSM, trans and gender‐diverse people.	[27, 36]
	HIVST is usable for GBMSM, trans and gender‐diverse people, including those who are marginalized based on ethnicity, education, sexual orientation, gender and migration status.	[27, 34, 35, 38, 53]
Values and preferences	Fourth‐generation tests strongly preferred because of short window period. For many, HIVST will be supplementary until this is available.	[37]
	MSM generally prefer blood‐based samples because of accuracy concerns, a minority with aversion to blood draw will not test using a blood‐based sample.	[34, 37, 57]
	MSM generally prefer postal delivered self‐testing for convenience. Those living in shared accommodation and with domestic privacy concerns (including many from ethnic minority backgrounds) require alternative to postal delivery options.	[37]
	Both written and video instruction options are useful.	[34, 37]
	Individuals with mild learning difficulties may benefit from additional support when using HIVST.	[62]
HIVST outcomes		
Testing uptake/frequency	HIVST can lead to dramatic increases in testing uptake and frequency among GBMSM, trans and gender‐diverse people by reducing barriers related to privacy, fear, opportunity cost, stigma and discrimination.	[19, 26, 27, 35, 36, 61, 65]
Positivity	HIVST can potentially lead to increases in numbers of diagnoses, especially in high prevalence populations.	[19]
Linkage to prevention	HIVST can reduce barriers to clinic attendance. For some SELPHI participants, re‐engaging with clinics through HIVST facilitated PrEP uptake.	[34, 40]
Linkage to care	Most people with positive results link to care quickly with support from kits and social networks.	[26, 34, 65]
	Linkage overall may be sub‐optimal compared to clinic‐based approaches for key populations overall (results not statistically significant for MSM and trans people).	[26, 34, 65]
STI testing	HIVST does not lead to statistically significant decreases in STI testing overall.	[26, 34, 65]
	Individuals who have high psychosocial or geographic barriers to testing may use HIVST as a primary testing method and test for STIs less frequently if not offered together.	[34, 40]
Harms	Harms from HIVST are very rare.	[19, 39, 64]
	Except for those relating to harms from the kit itself (false positives/negatives), harms do not usually reduce HIVST acceptability.	[39, 64]
Support	Most individuals access support (following both positive and negative results) from their wider social networks.	[34, 49]
	Those who have lack social network support will require additional supportive options when using HIVST.	[49]
Wider benefits	HIVST can increase confidence by providing an empowering HIV testing mechanism controlled by the beneficiary.	[34, 40]
	HIVST can facilitate first time or reengagement with sexual health services (including STI testing and HIV prevention) among those who do not have a pre‐existing relationship to sexual health services.	[40]

Abbreviations: GBMSM, gay, bisexual and other men who have sex with men; HIVST, HIV self‐testing; MSM, men who have sex with men; PrEP, pre‐exposure prophylaxis; SELPHI, HIV self‐testing public health intervention; STI, sexually transmitted infection.

### The HIVST Implementation Action Framework

#### Context of implementation

The wider public health drive towards HIV elimination is highly favourable for HIVST implementation. This includes the ‘Getting to Zero’ aim, which is frequently cited by public health bodies, the government as well as clinical and voluntary sector organizations [42].

A key component of ‘Getting to Zero’ is the 95–95–95 initiative, whereby 95% of people living with HIV know their status, 95% of those are on antiretroviral treatment and 95% of those have an undetectable viral load. HIV testing is the first step on this pathway. As testing options have expanded over time, the proportion of GBMSM with undiagnosed HIV has decreased. These decreases are not shared equitably, and marginalized groups continue to face higher burdens of HIV, as described previously [10]. In addition, trans and gender‐diverse people experience pronounced HIV testing need, due in part to a general lack of gender affirming sexual health services [3, 36]. As well as increasing the volume of testing, HIVST may also help to achieve health equity if targeted properly and also has the benefit of being cheaper than many other testing options. Improving equity should be a central consideration in implementation and is a key mechanism through which to argue for funding from commissioners. This, however, will require continued political support for the maintenance and expansion of HIV testing in an environment which is increasingly economically strained. Highlighting that HIVST can contribute to the continued expansion of testing in a financial sustainable way may be effective.

However, despite several notable successful interventions [31, 43, 44], and a wide range of evidence supporting HIVST, scale‐up has been hindered by various factors in the United Kingdom. Commissioner and policy makers' primary approach to self‐directed ‘home’ testing [45], thus far has been to provide HIVSS at scale through an online postal delivery. Indeed, HIVSS became widespread during the COVID‐19 pandemic and is now one of the main ways for HIV testing in many areas in England and Wales.

HIVSS has many benefits, including giving service users greater control over testing and clear pathways to support and follow‐up. HIVSS has limitations with sample return rates remaining low at between 50% and 60% [14, 15], largely driven by the difficulty of collecting sufficient blood. HIVST performs better with 90%–95% of individuals reporting their results [26, 27]. This means that the reliance on self‐sampling only may lead to missed testing and diagnosis opportunities. When service beneficiaries are offered a choice between HIVST or HIVSS, typically two‐thirds choose self‐testing, indicating service provision is not currently meeting user preferences [29, 30, 43].

A key implementation barrier for policymakers and commissioners is a concern that providing HIVST disconnects testing from HIV services which could lead to fewer people linking to care or being included in surveillance data [46, 47]. Evidence on this is mixed. A large randomized controlled trial (RCT) in England and Wales found no differences in linkage to care following a reactive HIVST result; however, a global meta‐analysis of HIVST interventions delivered to key populations in general (including in LMIC settings) found decreases in linkage to care overall, though results were not statistically significant for GBMSM and trans women (see Table 2) [19, 26, 28].

The potential of extensive HIVST implementation challenges us to think in new ways about choice and autonomy. To meet the needs of the broadest group of people—including the most marginalized—a diverse range of HIV testing interventions should be available to ensure that no one is left behind in the drive towards HIV elimination. Rather than relying on a narrow set of testing interventions, flexible services should be designed to meet a range of needs, including widespread access to HIVST. This echoes similar calls for more equitable HIV pre‐exposure prophylaxis (PrEP) services that reduce barriers faced by the most impacted groups (for example Asian, Black and younger GBMSM) who are currently underserved by implementation [48].

##### Intervention core components

###### Which kit to use

There are currently four HIVST kits available in the United Kingdom. Three of these use a whole blood sample (*SURE CHECK®, INSTI® and Mylan*) and one uses oral fluid (*OraQuick®*). All are second‐ or third‐generation which means they rely on detection of HIV1/2 antibodies and do not include antigen detection so are less useful in identifying very recent HIV acquisition (i.e., less than 4 weeks).

There are fourth‐generation RDTs available; however, none have yet been developed into an HIVST. Because these fourth‐generation RDTs detect both HIV antibody and antigen, they have a shorter window period and identify earlier infections. When they become available as self‐tests, they will become the gold standard because of a user preference for tests with a shorter window period [37]. Table 3 provides an overview of currently available tests in the United Kingdom, their sensitivity, specificity and various benefits.

**TABLE 3:** HIV self‐tests in the United Kingdom.

	Sample type	Generation	Sensitivity	Specificity	Window period	Benefits
OraQuick HIV self‐test	Oral fluid	Second	92%	99.98%	6 weeks (can take up to 12 weeks)	Simple to use
SureCheck HIV self‐test Chembio	Whole blood	Second	99.7%	99.9%	6 weeks (can take up to 12 weeks)	Simple to use
Insti rapid detection HIV self‐test	Whole blood	Third	100%	99.5%	4 weeks (can take up to 12 weeks)	Shorter window period
Mylan HIV self‐test	Whole blood	Third	99.6%	99.8%	4 weeks (can take up to 12 weeks)	Shorter window period

Generally, HIVST kits available in the United Kingdom are either very simple to use but with a longer window period, or more challenging to use but with a shorter window period. Decisions around which test(s) are implemented should be based on the trade‐off between higher performance and ease of use.

A third‐generation test using a whole blood sample will likely remain the basis of any HIVST services in the United Kingdom, due to the shorter window period and higher sensitivity than other tests, especially compared to a saliva‐based HIVST (e.g., OraQuick). However, services should consider having a secondary option providing oral fluid‐based HIVST for individuals who have concerns about or are unable to collect a blood sample and/or a phobia of needles or blood. If accessing an oral HIVST, supportive information should highlight that blood‐based kits have higher sensitivity and specificity. This is an especially important consideration for people with recent risk exposure or those using PrEP for whom detectable seroconversion can be blunted with oral fluid testing. Providing clear information during test selection will enable beneficiaries to decide which test is right for them.

###### Standard level of support

How to support people self‐testing is key to improving outcomes and well‐being among service beneficiaries. This may be a particular issue with HIVST where linkage to care could be sub‐optimal compared to clinic‐based testing [19].

We have developed a standard level of support (see Table 4) that should be provided with all HIVST interventions. This will ensure consistency across services so that beneficiaries know what to expect, irrespective of the test provided, how it is delivered and who runs the service. For most programmes, all components can be provided online and automated through the mechanisms individuals use to sign up to services.

**TABLE 4:** Standard level of support.

Component	Target group	Aim
Results reporting system	All intervention beneficiaries	Ensure data are available on proportions who have positive and negative HIV self‐test results Identify issues with kit delivery or performance Facilitate linkage to care for those with positive results
Linkage to prevention/care pathways	All intervention beneficiaries	Highlight other potential HIV prevention opportunities Facilitate linkage to care for those with positive results Facilitate linkage to PrEP for those testing negative
Helpline	All intervention beneficiaries	Provide access of support for those without supportive social networks Facilitate linkage to care for those with positive results
Clinical follow‐up	Intervention beneficiaries with positive results and with safeguarding concerns	Facilitate linkage to care for those with positive results Respond to identified safeguarding concerns

Abbreviation: PrEP, pre‐exposure prophylaxis.

For interventions designed to engage GBMSM, trans and gender‐diverse people with the highest privacy barriers (e.g., services delivering HIVST through peers, outreach, community‐based organizations and pharmacies), linkage to care information can be provided in physical form. This will usually be with web‐links or QR codes on a card packaged within the kit, eliminating the requirement for individuals to provide contact details.

###### Results reporting system

A further core intervention component for HIVST service delivery is having a mechanism for service users to voluntarily report their results. This enables follow‐up including facilitating linkage to care if required, identification of potential testing issues, and provide a mechanism to replace self‐tests if needed. A standard level of support should ideally have an active result reporting mechanism as default, meaning that the service should contact the beneficiary and ask them about their result, usually 8–14 days after their test was dispatched or collected. This will usually be done through an automated survey delivered via SMS, email or instant messaging (e.g., WhatsApp). Reminders should be used conservatively.

However, enabling individuals enrolling in an HIVST service to *opt‐out* of results reporting if they wish to do so will facilitate engagement with individuals with very high privacy barriers, ensuring widespread intervention acceptability while also providing protection from accidental disclosure from having messages on one's phone. Providing information on linkage to care and a passive result reporting system in physical form, potentially using a QR code delivered with the kit, can help facilitate uptake of support as required.

To specifically engage those with the greatest privacy concerns for whom providing contact details will reduce intervention acceptability substantially, distribution of self‐tests through outreach, peers or community‐based organizations is also advised. For such interventions, passive results reporting can be used, again using a QR code on accompanying supportive information. This approach may result in fewer individuals reporting their results, but will represent an important part of differentiated HIVST service delivery by explicitly attempting to reach those with the greatest privacy concerns.

###### Linkage to care pathways

Ensuring individuals can access care following HIVST is vital for connecting people with a positive (reactive) self‐test to confirmatory testing and treatment, and to link service beneficiaries with negative self‐test results to further support (e.g., sexually transmitted infections [STI] testing, HIV PrEP, doxyPEP, MenB and other vaccinations as well as other holistic sexual health services). Accompanying information should stress the critical importance of seeking follow‐up testing in a clinic to confirm any positive (reactive) HIVST results. Care pathway access can also be highlighted in communication about self‐testing following service enrolment.

###### Helpline

Most people who use HIVST seek support from their social networks [34, 39, 49]. However, a helpline should be available for those using HIVST who require immediate or confidential support, including individuals who do not have supportive social networks [49]. This can be bespoke and run by the implementing organization and link to existing services. Helpline details can be included in service communications, and with the kit itself. Hours of operation for the helpline should be clearly presented as it may inform when people use the test, particularly if they have concerns about a reactive result.

##### Potential intervention adaptations

A primary benefit of HIVST is the flexibility afforded by the intervention. Indeed, this type of testing can be packaged and delivered in a variety of ways. Choosing how to implement HIVST will require identifying the priority target population, how best to reach them and their likely support needs following self‐testing. In this section, we outline some of the intervention adaptations which can be made and which groups they support and how. This can be considered a menu of options available to service providers (see Table 5).

**TABLE 5:** HIVST intervention adaptations.

Component	Possible adaptations	Target audience	Evidence source
Delivery mode	**Postal**	**Most beneficiaries**	[19, 23, 27, 29, 31, 34, 37, 58]
	Click‐and‐collect from a postal locker	Beneficiaries with domestic privacy concerns	[23, 31, 37]
	Community‐based organization, pharmacy	Beneficiaries with capability or support needs	[19]
	Peer/partner delivered	Beneficiaries at higher risk	[19]
	Outreach	Beneficiaries at higher risk Beneficiaries with capability concerns Beneficiaries from ethnic minority backgrounds	[19]
	Other (e.g., vending machine, SOPV pick‐up)	Beneficiaries with high privacy barriers Beneficiaries at increased risk	[52]
Instructions	**Manufacturer's instructions**	**Most beneficiaries**	[19, 23, 27, 29, 31, 34, 37, 38, 58]
	Video	Most beneficiaries	[34, 37]
	Enhanced written instructions	Beneficiaries with minor capability needs	[37]
	Demonstration (in person/online)	Beneficiaries with pronounced capability needs, including mild learning disability	[62]
Accompaniments	**Linkage to testing and prevention**	**Beneficiaries from marginalized backgrounds** **Beneficiaries estranged from services**	[34, 40]
	Bacterial STI self‐sampling	Beneficiaries who test at clinics infrequently	[34, 40]
	Syphilis self‐testing	Beneficiaries who test at clinics infrequently and without prior syphilis diagnosis	[66, 67]
	HCV self‐testing	Beneficiaries without previous HCV diagnosis Beneficiaries engaged in chemsex and group sex	[55]
Demand generation	**Marketing**	**All beneficiaries**	[4, 27, 34, 40, 61]
	Risk assessments	All beneficiaries	[34, 40]
	Testing reminders	Beneficiaries who do not have a testing routine	[34, 40]
Support	**Results reporting**	**All beneficiaries**	[19, 23, 26, 27, 29, 31, 34, 36, 37, 58, 65]
	**Linkage to care pathways**	**All beneficiaries**	[19, 23, 26, 27, 29, 31, 34, 36, 37, 58, 65]
	**Help line**	**All beneficiaries**	[19, 23, 26, 27, 29, 31, 34, 36, 37, 58, 65]
	**Clinical follow‐up**	**Beneficiaries with positive results** **Beneficiaries with safeguarding issues**	[26, 29]
	Online counselling	Beneficiaries with highest needs, including learning disability	[49, 68, 69]

*Note*: Bold items are likely to be standard approaches in the United Kingdom.

Abbreviations: HCV, hepatitis C virus; SOPV, sex on premises venue; STI, sexually transmitted infection.

#### Delivery mode

Delivery mode is possibly the most important element of an HIVST service as it defines how scalable an intervention is and who is most likely to access it. Because self‐testing is so flexible, delivery options are varied. This guidance is not exhaustive but is intended to provide a range of options to consider based on the needs of the groups they are attempting to engage.

Online ordering and postal delivery will likely remain the cornerstone of HIVST service delivery. As GBMSM, trans and gender‐diverse people are usually a highly digitally literate population [50, 51], this approach has the benefit of having a low barrier to access and is straightforward for service providers to scale up. Postal delivery also enhances the convenience of HIVST while keeping costs low. Services which seek to reach large numbers should use this approach as a primary option, but acknowledge that it has limitations for the most marginalized, including GBMSM, trans and gender‐diverse people between the ages of 16 and 24, some individuals from ethnic minority communities, individuals with domestic privacy concerns and people who have no fixed address. Click‐and‐collect, whereby packages are sent to lockers and individuals type in a code to retrieve them, are especially useful in reaching these individuals.

Vending machine‐delivered HIVST may also be useful in reaching those with high privacy barriers and/or individuals at potentially increased risk in environments where they may socialize [52]. It should be noted that this is likely to be a very high‐cost option and only used in circumstances where there are a large number of GBMSM, trans and/or gender‐diverse people in one environment, such as sex‐on‐premises venues.

Community‐based organization and pharmacy‐distributed HIVST may be an effective way of delivering a service to those who have specific support concerns, including questions about test performance, or those with capability concerns and who require some guidance. Distributing self‐tests through these services will also help to reach the small minority who are not digitally literate.

Outreach workers can deliver self‐tests to their contacts while providing support in social and community venues. Peer/partner delivered HIVST is when an index person is provided with a number of self‐tests to distribute in their social networks. Both these approaches may have the benefit of reaching beneficiaries at increased risk and/or with privacy concerns who might not otherwise access testing. These models may facilitate engagement from poorly served GBMSM, trans and gender‐diverse people who face very high barriers to service access, including issues with digital literacy and experience of intense marginalization. Because these interventions are dislocated from clinical services, those implementing should be mindful of ensuring accompanying supportive information is comprehensive with clear links to follow‐on care, including additional HIV/STI testing and prevention opportunities.

#### HIVST kit instructions

All HIVST available commercially in the United Kingdom have manufacturer‐provided written and video instructions. Evidence suggests that these will be sufficient for the majority of beneficiaries to operate the tests successfully, especially with increasing experience [34]. Research has found that manufacturer‐provided instructions were sufficient for those experiencing multiple types of marginalization including at the intersections of education, gender, sexual orientation and ethnicity [53, 54]. However, should a service seek to engage those with enhanced capability concerns, such as those with language barriers or specific learning difficulties, more detailed instructions can be developed, ideally with the input of community members. As with all supportive information, the need to seek further facility‐based testing following reactive HIVST results should be emphasized.

#### Potential accompanying tests

The majority of interventions in the United Kingdom have provided HIVST without other accompanying tests. While evidence from a large online HIVST recruiting over 10,000 GBMSM and trans people shows that STI testing rates are not substantially impacted by HIVST [26, 28], it may be preferable, and more convenient, to provide HIVST packaged with STI test kits, as are currently provided through most HIVSS interventions.

Bacterial STI self‐sampling is easy for most people to do and can be distributed with HIVST, with samples returned to the lab and results provided later. Syphilis self‐testing is a relatively recent innovation and there is a UK‐approved syphilis antibody self‐test which could be provided alongside HIVST. However, because syphilis antibodies persist after treatment, these tests are only useful for those who have not previously had a syphilis diagnosis. Supporting information at service enrolment and accompanying the test must make this very clear.

Hepatitis C self‐testing using oral fluid has been approved by the WHO. While the test is not currently approved in the United Kingdom, it may be in the future. Providing hepatitis C self‐testing with HIVST would benefit beneficiaries at increased hepatitis C risk, including those who engage in chemsex, group sex or who inject drugs [55]. These tests also only detect antibodies, which stay in the blood following spontaneous clearance or successful treatment. Similar to syphilis self‐testing, accompanying information must underline that a positive hepatitis C self‐test does not necessarily indicate an active infection [55].

#### Demand generation

Engaging people in interventions is critically important. In most cases, HIVST service delivery will be accompanied by marketing. This should be responsive to the populations the intervention is seeking to reach, and highlight facilitators (e.g., convenience, ease of use, confidence) while reducing barriers (e.g., capability concerns, blood draw). Personal perspectives from diverse individuals who have accessed HIVST can be used to promote self‐testing to wide audiences. These initiatives should focus on reaching GBMSM, trans and gender‐diverse people in the online and physical spaces they socialize in, including social media and dating apps as well as bricks and mortar venues.

Testing reminders (messages sent to prompt ordering further tests) are a powerful tool to engage service beneficiaries in testing [34, 40]. With beneficiary opt‐in and consent, these can be delivered to the contact details provided when individuals sign up for HIVST. Service providers can also give beneficiaries the option for how frequently they would like to receive these reminders, based on their own testing routines, preferences and anticipated sexual behaviour. This is especially useful for those at increased risk and those who may have multiple competing personal pressures related to socio‐economic difficulties who may find it difficult to prioritize testing.

#### Additional support options

For the majority of HIVST users, the basic level of support outlined above will be sufficient to use HIVST safely and effectively, including for many people testing for the first time. However, some services, especially those reaching individuals with mild learning disability or profound capability/support concerns, may wish to establish an option for real‐time online counselling. This could draw on principles of pre‐ and post‐test counselling used in HIV testing and also provide practical support on kit usage. Another option is to deliver self‐testing in a location where healthcare workers are present (such as a community‐based organization) where practical or emotional support can be offered as needed.

In addition, linkage to other prevention opportunities can be provided alongside HIVST. This can include contact details to arrange follow‐up testing, PrEP initiation, doxy PEP, MenB and other vaccination, a direct booking link to a clinic or a link to outreach workers who can support onward referral. This may be especially useful for beneficiaries who face multiple forms of marginalization.

##### Organizations and individuals involved with implementation

As with all new interventions, in order to successfully implement HIVST, high‐level support from commissioning, service delivery and public health organizations will be key. HIVST may also be challenging for some organizations to support as it requires a paradigm shift, with implementing organizations relinquishing control to the service user, especially around decision‐making following (self)‐testing. Difficulties accepting this paradigm may be heightened by wider opposition to HIVST within the sector, especially organizations which may have divergent values and preferences around intervention delivery or are currently commissioned to deliver facility based or self‐sampling HIV testing options. This may especially be challenging with a national service which, by design, is less able to be attentive to local contexts [56].

For organizations involved in delivering HIVST, identification and support of ‘implementation champions’ may support effective service delivery. These individuals typically promote implementation of an intervention within organizations, and with collaborators involved in service delivery [65]. Ensuring these ‘champions’ are up to date on the evidence supporting HIVST implementation is critical. They can also be centrally involved in discussion with collaborating organizations that have different values and influence more broadly across the sector.

Wherever possible, within organizations, the value of HIVST programmes in facilitating positive outcomes for end users should be highlighted. Communication around this should draw on the evidence base, especially as it pertains to populations served by the organizations. Implementors can also highlight that self‐testing can free up organizational capacity to focus on those who have the greatest needs, improving health equity and creating new organizational opportunities. Finally, implementers can highlight how HIVST can further agency and self‐care among those who use it, a key facilitator for service providers [56].

##### Process of implementation

Successful person‐centred implementation requires considered stages; the full Implementation Action Framework provides guidance for four steps of implementation (Planning, Engaging, Implementing and Evaluating) for future HIVST interventions and outlines what organizations may want to consider during each step [23]. Evaluation is essential to improve services and demonstrate value for money and can be conducted at various points through the implementation. Formative evaluation is conducted early in the implementation of novel services or targeting new groups to refine interventions and ensure they are meeting client needs. Summative evaluation is useful in ensuring any HIVST intervention meets its own aims including reach, linkage to HIV prevention services for those testing negative and to HIV care for those with reactive results. Although harms related to HIVST are very rare [20, 44], it is essential to have a mechanism whereby individuals can report harms either from the kit itself or socially emergent harms relating to the social circumstances of individuals. More guidance on the process of implementation and potentially useful tools are available in the open access report on this Framework [23].

## CONCLUSIONS

HIVST is extremely flexible and can be packaged and delivered in myriad ways to address health inequalities. Reaching the full potential of HIVST encourages us to think granularly about how to reduce barriers for those with the greatest need such as trans and gender‐diverse people, GBMSM from minority ethnic backgrounds, individuals without supportive social networks and those experiencing multiple marginalization [35, 36, 49, 53]. HIVST can complement facility‐based HIV testing services by offering an additional highly acceptable and cost‐effective testing option with less opportunity cost and wider geographical reach, and by engaging new audiences with HIV testing for the first time. When integrated within a constellation of sexual health services, HIVST has the potential to reduce health inequalities particularly among GBMSM, trans and gender‐diverse people.

Through evidence synthesis and community and sectoral engagement, we produced this Implementation Action Framework and Tool‐Kit [22] as a resource for those seeking to plan, implement and evaluate HIVST among GBMSM, trans and gender‐diverse people in the United Kingdom. The guidance is not meant to be prescriptive but provides an implementation roadmap detailing innovative approaches and the evidence underpinning them that can be used to improve health equity among the most marginalized populations at risk of HIV acquisition.

## AUTHOR CONTRIBUTIONS

Funding: AJR, TCW, PW, FMB. Conceptualisation: TCW, AJR. Analysis: TCW. Critical input: TCW, AJR, PW, PS, FMB, IYHC. Drafting: TCW, AJR. Editing: ARJ, PW, FMB, PS, IYHC.

## FUNDING INFORMATION

This manuscript presents independent research funded by the National Institute for Health and Care Research (NIHR) under the Programme Development Grants (Reference number: NIHR203298). The views expressed in this manuscript are those of the author(s) and not necessarily those of the NHS, the National Institute for Health Research or the Department of Health and Social Care.

## CONFLICT OF INTEREST STATEMENT

TCW received speaking honoraria from Gilead Sciences between 2017 and 2023. The remaining authors declare no conflicts of interest.

## Data Availability

Data sharing not applicable to this article as no datasets were generated or analysed during the current study.
